# Effects of work organization on the occurrence and resolution of sleep disturbances among night shift workers: a longitudinal observational study

**DOI:** 10.1038/s41598-021-85017-8

**Published:** 2021-03-09

**Authors:** Seungho Lee, Jae Bum Park, Kyung-Jong Lee, Seunghon Ham, Inchul Jeong

**Affiliations:** 1 Department of Occupational and Environmental Medicine, Ajou University School of Medicine, 164 World cup-ro, Yeongtong-gu, Suwon, 16499 South Korea; 2 Department of Occupational and Environmental Medicine, Gil Medical Center, Gachon University College of Medicine, Incheon, 21565 South Korea

**Keywords:** Epidemiology, Sleep disorders

## Abstract

This study aimed to investigate the association between work organization and the trajectories of insomnia patterns among night shift workers in a hospital. The health examination data of hospital workers, recorded from January 2014 to December 2018, were collected; 6765 records of 2615 night shift workers were included. Insomnia was defined as a score of ≥ 15 on the Insomnia Severity Index (ISI). Participants were categorized into five groups according to insomnia patterns derived from the analysis of their ISI scores. Work organization and socio-demographic characteristics were also investigated. Generalized estimating equation models and linear mixed models were constructed to analyze the longitudinal data. Of the total participants, 53.0% reported insomnia at least once during the follow-up period. The lack of nap opportunities and work-time control was associated with the occurrence of insomnia, whereas more than 5 years of shift work experience was related to the resolution of insomnia. All work-related factors were significantly related to insomnia risk; however, the effects were not significant in the sustained insomnia group. Although sleep problems are inevitable in night shift workers, well-designed work schedules and better work organization can help reduce the occurrence of insomnia among them.

## Introduction

Shift work has become a common type of work schedule in many countries. It is reported that 18 percent of employees in EU work outside normal daytime hours^1^. Healthcare workers are known to commonly work in shifts; they make up the largest proportion of shift workers in many regions. It has been reported that approximately 40% of healthcare workers are shift workers^2,3^.

However, shift work can lead to several health problems including cardiovascular, gastrointestinal, and metabolic disorders. Furthermore, the relationships between shift work and cancer, mental disorders, and reproduction problems have been reported^4^. Several mechanisms, such as circadian disruption, disturbed sleep, behavioral change, psychosocial stress, and physiological change have been suggested as plausible reasons behind these relationships^4,5^. Among these possible mechanisms, occurrence of sleep disorders is one of the most important ones, since it is both an adverse health outcome of circadian disruption and an underlying cause of other health problems.

According to the International Classification of Sleep Disorders, 3rd edition, sleep disorder induced by shift work is called shift work disorder (SWD) and is characterized by complaints of insomnia and/or excessive sleepiness^6^. The risk of SWD varies according to many individual and work-related factors. A previous systematic review that included 85 studies concluded that individual factors, such as older age, morning-type personality, circadian flexibility, being married or having children, increased caffeine intake, higher neuroticism scores, and lower hardiness scores were related to a higher risk of SWD, whereas physical activity was a protective factor^7^. Another review that included 24 studies suggested that work demands, job strain, bullying, and effort-reward imbalance are related to more sleep disturbances, whereas social support at work, control, and organizational justice are related to fewer sleep disturbances^8^.

Notably, identifying the harmful work-related factor of shift work is as important as identifying the vulnerable populations. Several studies have reported the associations between quick return (11 or less hours of rest between shifts) and sleep disorders^9–11^. However, long-term impacts of other shift work-specific work organization on sleep problems have not been well established^7,12^. Furthermore, the number of longitudinal studies on the occurrence or resolution of insomnia is limited.

In the present study, we explored the impact of work organization on the trajectories of insomnia patterns among night shift workers. Using longitudinal health examination data, we categorized participants according to the insomnia patterns derived from the analysis of their Insomnia Severity Index (ISI) scores, and investigated the impacts of work organization, including both shift work-specific and other work-related factors that are known to cause sleep problems^13–15^, on the occurrence and the resolution of insomnia.

## Results

**Figure 1:**
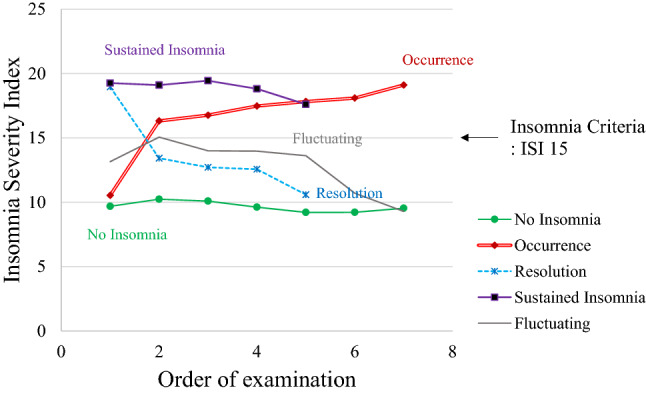
Insomnia patterns recorded during the study period. *ISI* Insomnia Severity Index.

The study consisted of 606 male (23.2%) and 2009 female (76.8%) participants, with a mean age of 27.9 ± 6.42 years (range 21.0–59.0 years). The 2615 night shift workers evaluated in the present study were retrospectively followed-up seven times. Table 1 shows that 53.0% of the participants reported sleep disturbances at least once during the follow-up period. More than half the participants had shift work experience of < 5 years (N = 1735, 66.4%), no nap opportunities (N = 2080, 79.5%), and no work-time control (N = 1432, 54.8%). Furthermore, 812 (31.1%) participants reported quick return, whereas 862 (33.0%) reported insomnia at baseline (Table 1). Regarding night shifts and working hours, the proportions of participants who had consecutive night shifts for 3 days (N = 1215, 46.5%) and weekly working hours of 41–51 h (N = 947, 36.2%) were the highest. Figure 1 presents the insomnia patterns of the participants during the study period according the defined groups using least square means. The distribution of participants according to the patterns of insomnia was as follows: 47.0% of the participants were included in the no insomnia group, 13.8% were included in the insomnia occurrence group, 11.5% were included in the insomnia resolution group, 18.1% were included in the sustained insomnia group, and 9.6% were included in the fluctuating insomnia group.

**Table 1:** General characteristics of the study participants at baseline.

	Male (n = 606)		Female (n = 2009)		Total (n = 2615)	
	N	(%)	N	(%)	N	(%)
Age, mean (range)	30.6	(23.0, 59.0)	27.1	(21.0, 59.0)	27.9	(21.0, 59.0)
BMI, mean (range)	24.4	(16.5, 37.4)	21.4	(14.2, 37.4)	22.1	(14.2, 37.4)
**Smoke**						
Yes	136	(22.4)	2	(0.10)	138	(5.28)
No	470	(77.6)	2007	(99.9)	2477	(94.7)
**Drink**						
< 1/week	180	(29.7)	1008	(50.2)	1188	(45.4)
1–2/week	374	(61.7)	911	(45.4)	1285	(49.1)
3–7/week	52	(8.58)	90	(4.48)	142	(5.43)
**ISI score**						
0–7	461	(76.1)	1292	(64.3)	1753	(67.0)
8–14	124	(20.5)	563	(28.0)	687	(26.3)
15–21	18	(2.97)	135	(6.72)	153	(5.85)
22–28	3	(0.50)	19	(0.95)	22	(0.84)
**Shift work experience**						
Never	45	(7.43)	230	(11.5)	275	(10.5)
< 5 years	474	(78.2)	1261	(62.8)	1735	(66.4)
5–9 years	33	(5.45)	289	(14.4)	322	(12.3)
10–14 years	12	(1.98)	136	(6.77)	148	(5.66)
15–19 years	16	(2.64)	82	(4.08)	98	(3.75)
≥ 20 years	26	(4.29)	11	(0.55)	37	(1.41)
**Quick return**						
Yes	240	(39.6)	572	(28.5)	812	(31.1)
No	366	(60.4)	1437	(71.5)	1803	(69.0)
**Consecutive night shift**						
1 day	275	(45.4)	443	(22.1)	718	(27.5)
2 days	99	(16.3)	226	(11.3)	325	(12.4)
3 days	136	(22.4)	1079	(53.7)	1215	(46.5)
4 days	16	(2.64)	44	(2.19)	60	(2.29)
≥ 5 days	80	(13.2)	217	(10.8)	297	(11.4)
**Nap opportunity**						
Yes	286	(47.2)	249	(12.4)	535	(20.5)
No	320	(52.8)	1760	(87.6)	2080	(79.5)
**Work-time control**						
Yes	389	(64.2)	794	(39.5)	1183	(45.2)
No	217	(35.8)	1215	(60.5)	1432	(54.8)
**Weekly working hours**						
< 40 h	45	(7.43)	160	(7.96)	205	(7.84)
40 h	122	(20.1)	630	(31.4)	752	(28.8)
41–51 h	144	(23.8)	803	(40.0)	947	(36.2)
52–59 h	61	(10.1)	224	(11.2)	285	(10.9)
≥ 60 h	234	(38.6)	192	(9.56)	426	(16.3)

Overall, the latest ISI score was significantly higher than the initial ISI score by 0.28, and the intra-individual differences were significant (*p* = 0.0473). Each work-related factors significantly contributed to the differences, except quick return. Interestingly, the ISI increased 1.91 points among those who reported less than 5 years of shift work experience; however, the score decreased among those who reported more than 5 years of shift work experience (*p*-value for intra- and inter-individual effects: < 0.0001). In addition, the ISI increased in all three groups with over 40 working hours per week (*p* = 0.0001) (Table 2).

**Table 2:** Mean differences between the first and last Insomnia Severity Index scores stratified according to work organization.

	N	First ISI–Last ISI			*p* value	
		Mean difference	Confidence interval		Within	Between
All	1747	− 0.28	− 0.56	0.00	0.0473	–
**Shift work experience**						
Never	4	− 2.00	− 6.68	2.68	< 0.0001	< 0.0001
< 5 years	1029	− 1.91	− 2.24	− 1.58		
5–9 years	343	2.02	1.40	2.63		
10–14 years	193	2.45	1.61	3.29		
15–19 years	98	2.33	1.02	3.63		
≥ 20 years	80	1.10	0.11	2.09		
**Quick return**						
Yes	522	− 0.64	− 1.13	− 0.15	0.0964	0.3003
No	1225	− 0.13	− 0.46	0.21		
**Consecutive night shift**						
1 day	259	0.58	0.01	1.15	0.0512	< 0.0001
2 days	202	0.10	− 0.69	0.90		
3 days	1027	− 0.57	− 0.95	− 0.19		
4 days	36	− 0.53	− 2.56	1.50		
≥ 5 days	223	-0.28	-1.05	0.49		
**Nap opportunity**						
Yes	312	− 0.01	− 0.53	0.51	0.3716	< 0.0001
No	1435	− 0.34	− 0.66	− 0.02		
**Work-time control**						
Yes	486	0.15	− 0.31	0.60	0.0611	< 0.0001
No	1261	− 0.45	− 0.79	− 0.10		
**Weekly working hour**						
< 40 h	58	0.28	− 1.28	1.83	0.039	0.0001
40 h	345	0.41	− 0.22	1.04		
41–51 h	765	− 0.30	− 0.73	0.13		
52–59 h	243	− 1.35	− 2.16	− 0.54		
≥ 60 h	336	− 0.27	− 0.81	0.27		

Table 3 shows the results of the generalized estimating equation (GEE) model for the effects of work organization on insomnia. All factors were significantly related to insomnia after adjusting for sex, age, body mass index (BMI), smoking, and drinking. The odds ratio (OR) for insomnia among participants who had shift work experience of 5–9 years (OR 3.69, 95% CI 2.34–5.84) was significantly higher than that of those who had no night shift work experience. Quick return, nap opportunity, and work-time control were associated with 13% (95% CI 1.01–1.28), 24% (95% CI 1.01–1.52), and 70% (95% CI 1.49–1.94) higher odds of insomnia, respectively. Participants who worked consecutive night shifts had about two-fold higher odds of insomnia than those who worked a one-day night shift, and those who worked over 40 h per week had about two-fold higher odds of insomnia than those who worked less than 40 h per week.

**Table 3:** Longitudinal effects of work organization on insomnia.

Source	All participants			Insomnia occurrence			Insomnia resolution		
	OR estimates	Confidence interval	*p *value	OR estimates	Confidence interval	*p *value	OR estimates	Confidence interval	*p *value
**Shift work experience**									
Never	1	Reference	< 0.0001	1	Reference	0.0435	- *		
< 5 years	3.51	(2.28–5.40)		1.71	(1.20–2.43)		1	Reference	0.0066
5–9 years	3.69	(2.34–5.84)		1.46	(0.86–2.49)		1.64	(1.16–2.32)	
10–14 years	3.14	(1.90–5.18)		1.93	(0.96–3.89)		2.14	(1.25–3.68)	
15–19 years	2.76	(1.53–4.97)		1.41	(0.62–3.19)		1.32	(0.59–2.98)	
≥ 20 years	2.59	(1.32–5.06)		1.05	(0.34–3.20)		0.72	(0.22–2.36)	
**Quick return**									
No	1	Reference	0.039	1	Reference	0.7678	1.07	(0.84–1.36)	0.5784
Yes	1.13	(1.01–1.28)		1.03	(0.84–1.26)		1	Reference	
**Consecutive night shift**									
1 day	1	Reference	< 0.0001	1	Reference	0.05	0.64	(0.39–1.05)	0.3563
2 days	1.8	(1.45–2.24)		1.12	(0.81–1.55)		0.84	(0.53–1.34)	
3 days	2.11	(1.74–2.55)		1.46	(1.11–1.91)		0.91	(0.63–1.31)	
4 days	1.88	(1.32–2.67)		1.36	(0.80–2.32)		1.32	(0.62–2.77)	
≥ 5 days	2.08	(1.68–2.59)		1.43	(1.01–2.03)		1	Reference	
**Nap opportunity**									
Yes	1	Reference	0.0362	1	Reference	0.0021	1.08	(0.68–1.74)	0.7372
No	1.24	(1.01–1.52)		1.65	(1.20–2.28)		1	Reference	
**Work-time control**									
Yes	1	Reference	< 0.0001	1	Reference	< .0001	1.14	(0.85–1.52)	0.3949
No	1.7	(1.49–1.94)		1.56	(1.27–1.91)		1	Reference	
**Weekly working hour**									
< 40 h	1	Reference	< 0.0001	1	Reference	0.9426	1.05	(0.46–2.39)	0.0166
40 h	1.17	(0.90–1.52)		0.98	(0.69–1.40)		0.7	(0.43–1.14)	
41–51 h	1.88	(1.45–2.43)		0.98	(0.68–1.40)		0.53	(0.34–0.81)	
52–59 h	1.96	(1.48–2.61)		1.1	(0.73–1.65)		0.64	(0.41–1.02)	
≥ 60 h	1.89	(1.42–2.54)		0.98	(0.63–1.51)		1	Reference	

The generalized estimating equation model includes age, sex, body mass index, smoking status, and drinking status as covariates.

*Due to lack of power, 25 records reporting no shift work experience were not included in this analysis.

In the analysis of the occurrence of insomnia, shift work experience, consecutive night shifts, nap opportunity, and work-time control were significant risk factors. The risk was higher for those who had shift work experience of less than 5 years (OR 1.71, 95% CI 1.20–2.43). Further, nap opportunity and work-time control were associated with a 1.65-fold (95% CI 1.20–2.28) and 1.56-fold (95% CI 1.27–1.91) higher risk for the occurrence of insomnia, respectively. Meanwhile, the influential factor for resolution of insomnia was shift work experience. Instead of those who had no shift work experience, the participants who reported less than 5 years of experience were used as a reference group in the analysis for the resolution of insomnia. Participants who had shift work experience of 5–9 years (OR 1.64, 95% CI 1.16–2.32) and 10–14 years (OR 2.14, 95% CI 1.25–3.68) had higher odds of resolution than those with shift work experience of less than 5 years. Notably, there was no relationship between weekly working hours and resolution and occurrence of insomnia, even though the risk of occurrence of insomnia among the participants was significant.

**Figure 2:**
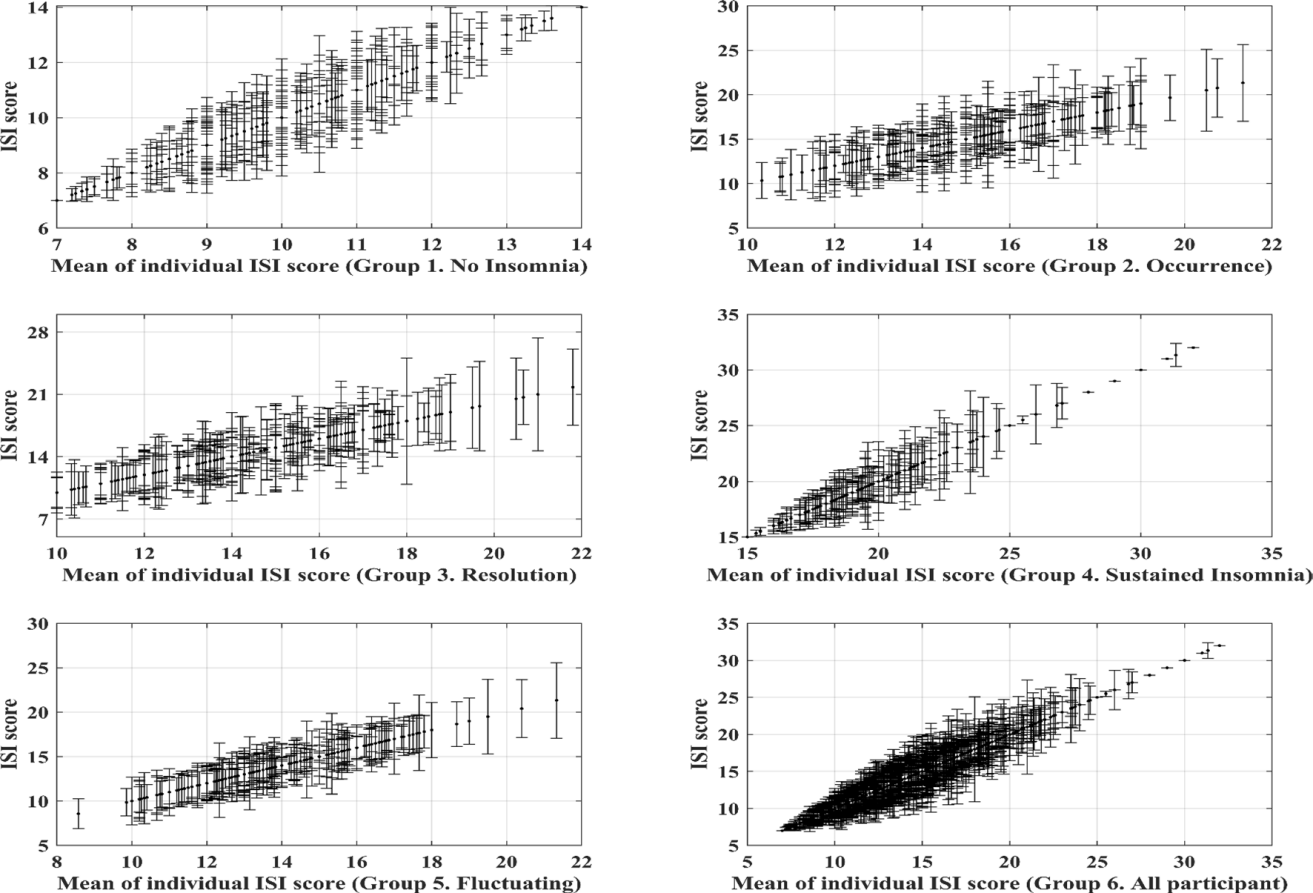
Variability of Insomnia Severity Index scores in each group. The *x*-axis indicates individual means, and the vertical lines indicate individual standard deviation of Insomnia Severity Index scores. *ISI* Insomnia Severity Index.

Figure 2 shows the variations in ISI scores by group, and each vertical line represents the range of ISI scores in a participant. The variations in ISI scores were small in the sustained insomnia group compared to those of the other groups. Notably, regarding the effects of work organization on ISI scores, all work related factors were not significant for the deterioration of ISI scores in the sustained insomnia group (Supplement Table). The global mean of the ISI scores was 17.46, and the effect of quick return, no nap opportunity, and no work-time control showed positive estimates. Additionally, the effects of > 40 working hours per week led to a decrease in ISI scores, but without any significance.

## Discussion

In this study, we investigated the association between work organization and the trajectories of insomnia patterns among night shift workers in a hospital. We noted that all work-related factors were significantly related to the risk of insomnia. The results suggest that night shift work induces sleep problems^16^; however, specific work-related factors can further deteriorate sleep health among night shift workers.

Generally, people who work long hours and have quick returns do not have enough time for rest, domestic work, or leisure; therefore, they perceive that their time is limited. It causes them to be preoccupied with sleep, try to sleep intentionally, and increase their sleep effort. According to the attention-intention-effort pathway, those reactions are important components in the development of insomnia^17^. A previous study showed a positive relationship between sleep effort and subjective insomnia^18^, and other studies reported results consistent with this finding as well^11,19^. However, in the present study, quick return was not significantly associated with the occurrence and resolution of insomnia, although the association with insomnia was significant in all participants. Regarding weekly working hours, the risk of insomnia generally increased as weekly working hours increased; however, it was not a significant factor for the occurrence or resolution of insomnia.

Additionally, disruption of circadian rhythm and melatonin regulation may have played a role in the observed relationship. The suppression of the amplitude of melatonin with increasing numbers of consecutive night shifts suggests that workers with more consecutive night shifts have more severe sleep problems^20^. In addition, nap opportunities may have affected the melatonin levels of the study participants. Although its impact on melatonin level was not conclusive in previous studies^21,22^, on-shift napping may reduce the duration of total light exposure at night^23,24^. As supportive evidence, no nap opportunity was one of the major risk factors for the occurrence of insomnia among those who did not have insomnia at baseline in the present study (OR for occurrence: 1.65, 95% CI 1.20–2.28).

Low work-time control is likely to cause poor recovery and work-family conflict, which are related to the occurrence of insomnia. Previous studies also indicated that low work-time control, long working hours, and unfavorable psychosocial work organizations are related to an increased risk for insomnia or future development of sleep disturbances^8,13–15^. In the present study, work-time control showed a significant association with the occurrence of insomnia among those who did not have insomnia at baseline (OR for occurrence 1.56, 95% CI 1.27–1.91), whereas the odds of resolution were not significant among those who had insomnia at baseline (Table 3).

Shift work experience was also related to an increased risk of insomnia in the present study. Our results consistently showed that insomnia was alleviated with increasing shift work experience. The OR of insomnia increased with increasing shift work experience up to 9 years of experience, and then decreased. Furthermore, less than 5 years of experience was associated with a 1.71-fold higher risk of occurrence of insomnia among those who reported no insomnia previously. Regarding those who had insomnia, 5–14 years of shift work experience was significantly associated with resolution of insomnia compared to less than 5 years of shift work experience (Table 3). These results demonstrated that 0–5 years of shift work experience is the major influential factor for occurrence and deterioration of insomnia.

We considered the healthy worker effect as an explanation for these findings^25^. Based on the last examination records of the participants, the prevalence of insomnia among the participants who dropped out during the follow-up period was slightly higher (41.1%) than that among the participants who completed the examination until 2018 (39.5%). Therefore, although the difference was small, there is a possibility that workers with sleep problems quit night shift work early in their career. A previous study reported findings similar to ours^26^.

As shown in Fig. 2, the variations of ISI scores in the sustained insomnia group were comparatively small. Therefore, we further analyzed the effects of work organization on the deterioration of ISI in the sustained insomnia group (see the Supplement Table). Interestingly, any work-related factors were not shown significance to the deterioration of ISI in the sustained insomnia group, whereas those relationships were significant for all participants. These results indicate that those who had insomnia consistently would have other reasons that contributed to their insomnia more than the individual- and work-related factors included in this study. In fact, there are a lot of factors that affect sleep disturbances, such as living patterns, psychosocial stress, health status, personal medications, and individual susceptibility. Thus, it is necessary to identify and compare the major contributing factors from various sources.

This study has several limitations. Firstly, the individual occupation records were not available, so the occupation effect for sleep disturbances could not be considered. Secondly, the study participants were mainly women and were from a single hospital. Therefore, we could not conclude whether the results of this study are generalizable to workers in other fields or male-dominant industry. Thirdly, insomnia was assessed based on the responses in a subjective questionnaire; details of actual sleep duration were not obtained. Therefore, although the ISI is a well-known validated instrument for screening insomnia, accurate diagnosis could not be achieved^27^. Fourthly, we could not assess other health conditions commonly comorbid with insomnia, such as psychiatric disorders, obstructive sleep apnea, and restless leg syndrome^28^. Also, effect of work-related stress were not assessed. Furthermore, some work-related factors were investigated according to “yes” or “no” responses. Therefore, investigating the dose–response relationship between quick return, nap, and insomnia was not possible. Lastly, the healthy worker effect may have impacted our results. Notably, the healthy worker effect may also have resulted in an underestimation of the effects of shift work. However, we used a longitudinal study design, and followed-up with the participants for up to 4 years. Therefore, recall bias or reverse causation was minimized in the present study. Additionally, the large number of participants is another strength of this study.

In conclusion, shift work is unavoidable for hospital workers in the modern society. However, the risk of insomnia among night shift workers varies according to work organization. Although sleep problems are inevitable for many night shift workers, the results of this study suggest that a well-designed work schedule and better work organization can help reduce the occurrence of insomnia among them. Work schedules that incorporate sufficient rest time, nap opportunities, and work-time control are required to prevent undesirable health outcomes related to insomnia. Furthermore, consecutive night shifts and long working hours should be limited. Future studies are required to examine other factors, including work organization, socio-demographic factors, and health status that contribute to insomnia.

## Methods

### Study participants

Under the Occupational Safety and Health Act of Korea, night workers are obligated to undergo an annual health examination. Night work is defined as follows: an 8-h duty including the hours from midnight to 05:00 h for an average of four times or more per month in the past 6 months, or 60 or more hours of work between 22:00 h to 06:00 h in the past 6 months^29^. For this study, we used the health examination data of night shift workers who were working in a university hospital from 2014 to 2018. A total of 7997 records of 3118 night shift workers were collected and analyzed. Cases with insufficient/no response records (878) and irregular night shift experiences due to department changes (354) were excluded. Finally, 6765 records of 2615 night shift workers were included in the present study. Informed consent was obtained from all participants, and the Institutional Review Board of Ajou University Hospital reviewed the protocol and approved this study (AJIRB-MED-MDB-18-154). This study was conducted in accordance with the STROBE guidelines.

### Insomnia, work-related factors, and other covariates

The health examination data analyzed in this study included demographic factors, anthropometric measures such as height and weight, and questionnaires on work organization, medical history, and insomnia. Among the investigated factors, sex, age, BMI, smoking history, and history of alcohol consumption were included in the analyses. Age and BMI were used as continuous variables, smoking history was divided into two groups according to the current smoking status (yes, no), and history of alcohol consumption was divided into three groups (< 1 time/week, 1–2 times/week, and 3–7 times/week). In addition, six components of work organization were included as follows: shift work experience (< 5, 5–9, 10–14, 15–19, ≥ 20 years, and never), quick return (yes, no), consecutive night shift (1, 2, 3, 4, and ≥ 5 days), nap opportunity (yes, no), work-time control (yes, no), and weekly working hours (< 40, 40, 41–51, 52–59, and ≥ 60 h).

Insomnia was assessed using the ISI, which consists of seven questions on difficulty in falling asleep, difficulty in staying asleep, problems waking up too early, sleep satisfaction, anxiety regarding insomnia, and interference with daily functioning experienced in the past 2 weeks. Each item is rated 0 (none), 1 (mild), 2 (moderate), 3 (severe), or 4 (very severe). The total score indicates severity of insomnia as follows: 1–7, no clinical significance; 8–14, subthreshold insomnia; 15–21, clinical insomnia (moderate); and 22–28, clinical insomnia (severe). In this study, we defined insomnia as having an ISI score of 15 or higher, according to the ISI author recommendations^30^.

### Participant grouping

The general characteristics and distributions of individual ISI scores were evaluated. Subsequently, we categorized the participants according to the insomnia patterns derived from the ISI scores. The *no insomnia* group included participants whose ISI scores were below 15 during the follow-up period (n = 1229). The *insomnia occurrence* group included participants whose ISI scores were below 15 at baseline, but insomnia occurred during the study period and persisted until the end of the follow-up period (n = 362). The *insomnia resolution* group included those who had insomnia at baseline, but the insomnia resolved at the end of the study (n = 301). The *sustained insomnia* group included participants whose ISI scores were 15 or more consistently from baseline to the end of the follow-up period (n = 472). The *fluctuating insomnia* group included those whose insomnia patterns did not match the definitions of the other four groups (n = 251).

### Statistical analysis

The repeated measure analysis of variance was used to analyze the effects of work organization while considering intra- and inter-individual effects. The GEE method was used to estimate the long-term effects of work organization on insomnia. The covariance structure specified compound symmetry, and the model included the six components of work organization: shift work, quick return, consecutive night shift, nap opportunity, work-time control, and weekly working hours, as well as age, sex, BMI, smoking status, and alcohol consumption status as covariates. The first analysis was conducted for all the participants. To evaluate the impact of work organization on the occurrence of insomnia, the second analysis was conducted for the no insomnia and insomnia occurrence groups. The third analysis was conducted for the sustained insomnia and insomnia resolution groups to investigate the effects of work organization on the resolution of insomnia. Due to lack of power, 25 records of participants that reported no shift work experience were not included in the third model. Lastly, a linear mixed model was used to discover the impact of work organization on the deterioration of ISI scores in the sustained insomnia group. This model included age, sex, BMI, smoking status, and alcohol consumption status as covariates, and compound symmetry was specified as the covariance structure. All statistical analyses were performed using SAS version 9.4 (SAS Institute, Cary, NC, US) and the trend of variability was figuring out using MATLAB R2020a, MathWorks, MA, US).

## Supplementary Information

Supplementary Information.

## Data Availability

Data are not available to public.
