# Supplementary material for: Effects of work organization on the occurrence and resolution of sleep disturbances among night shift workers: a longitudinal observational study
Source: Sci Rep. 2021 Mar 9;11:5499. doi: 10.1038/s41598-021-85017-8 (PMC7970909; doi:10.1038/s41598-021-85017-8)
Supplement: Supplementary file 1 — Supplementary Information. [file 41598_2021_85017_MOESM1_ESM.docx]

**Effects of work organization on the occurrence and resolution of sleep disorder among night shift workers: a longitudinal observational study**

Seungho Lee^1^, Jae Bum Park^1^, Kyung-Jong Lee^1^, Seunghon Ham^2^, Inchul Jeong ^1^

**Supplementary materials**

Table. Association between Insomnia Severity Index and work organization in the sustained insomnia group

Figure. Flowchart of participant selection

Supplementary Table. Association between Insomnia Severity Index and work organization in the sustained insomnia group

|  |  | **Mixed model** | | |
| --- | --- | --- | --- | --- |
| Source |  | Estimates | Standard Error | *p-value* |
| Intercept |  | 17.46 | 1.776 |  |
| Shift work | Never | - * | | 0.5329 |
| Experience | < 5 years | Reference | |  |
|  | 5–9 years | -0.008 | 0.330 |  |
|  | 10–14 years | 0.134 | 0.506 |  |
|  | 15–19 years | -0.342 | 0.757 |  |
|  | ≥ 20 years | 1.268 | 1.017 |  |
| Quick return | No | Reference | | 0.8412 |
|  | Yes | 0.054 | 0.268 |  |
| Consecutive | 1 day | Reference | | 0.6038 |
| Night shift | 2 days | -0.127 | 0.549 |  |
|  | 3 days | 0.184 | 0.467 |  |
|  | 4 days | -0.926 | 0.963 |  |
|  | ≥ 5 days | 0.367 | 0.519 |  |
| Nap Opportunity | Yes | Reference | | 0.3765 |
|  | No | 0.433 | 0.489 |  |
| Work-time | Yes | Reference | | 0.1114 |
| Control | No | 0.492 | 0.308 |  |
| Weekly | < 40 hours | Reference | | 0.7412 |
| Working hour | 40 hours | -0.539 | 0.888 |  |
|  | 41–51 hours | -0.268 | 0.865 |  |
|  | 52–59 hours | -0.046 | 0.903 |  |
|  | ≥ 60 hours | -0.505 | 0.941 |  |

* Due to lack of power, 25 records reporting no shift work experience were not included in this analysis.

The mixed model includes age, sex, body mass index, smoking status, and drinking status as covariates.

Special health examination data

N=3,118, number of records=7,997

Insufficient data or no response

N=408, number of records=878

Did not meet inclusion criteria

N=95, number of records=354

Study population

N=2,615, total number of records=6,765

2. Insomnia Occurrence

N=362, number of records=1,165

1. Insomnia

N=1229, number of records=2,426

3. Insomnia Resolution

N=301, number of records=1,095

4. Sustained Insomnia

N=472, number of records=971

5. Fluctuating insomnia

N=251, number of records=1,108

Supplementary figure. Flowchart of participant selection
